# Personality Traits, Education, Physical Exercise, and Childhood Neurological Function as Independent Predictors of Adult Obesity

**DOI:** 10.1371/journal.pone.0079586

**Published:** 2013-11-08

**Authors:** Helen Cheng, Adrian Furnham

**Affiliations:** 1 Department of Psychology, University College London, London, United Kingdom; 2 BI: Norwegian Business School, Oslo, Norway; Children's National Medical Center, Washington, United States of America

## Abstract

**Objective:**

To investigate whether personality traits, education, physical exercise, parental socio-economic conditions, and childhood neurological function are independently associated with obesity in 50 year old adults in a longitudinal birth cohort study.

**Method:**

The sample consisted of 5,921 participants born in Great Britain in 1958 and followed up at 7, 11, 33, 42, and 50 years with data on body mass index measured at 42 and 50 years.

**Results:**

There was an increase of adult obesity from 14.2% at age 42 to 23.6% at 50 years. Cohort members who were reported by teachers on overall clumsiness as “certainly applied” at age 7 were more likely to become obese at age 50. In addition, educational qualifications, traits Conscientiousness and Extraversion, psychological distress, and physical exercise were all significantly associated with adult obesity. The associations remained to be significant after controlling for birth weight and gestation, maternal and paternal BMI, childhood BMI, childhood intelligence and behavioural adjustment, as well as diet.

**Conclusion:**

Neurological function in childhood, education, trait Conscientiousness, and exercise were all significantly and independently associated with adult obesity, each explained unique individual variability.

## Introduction

 In the past two decades obesity in children and adults in developed countries has become an epidemic [1,2]. The consequences of adult obesity include health, social and economic consequences [3], as well as educational and psychological outcomes for individuals and their society [4]. 

 Previous studies on obesity have found an inverse relation between obesity and socioeconomic status [5], though the causal direction of this association is not clear. Some studies show that obesity may influence socioeconomic status due to discrimination in the work place (affecting promotion and earnings) and other social settings [6,7]. Other studies found evidence to suggest that socioeconomic conditions may influence obesity and poor health in general [8-10]. Previous research has shown that there are significant associations between childhood height and adult obesity [11], between parental obesity and BMI in adulthood [12], and between foetal and early life growth and BMI [13]. There is also evidence of links between depression and obesity [14], and between childhood intelligence and adult obesity controlling for diet [15]. 

One recent study showed that a set of poor neurological function measures (named poor hand control, poor coordination, clumsiness) assessed by teachers when cohort members were 7 year olds, were significantly associated with obesity in adulthood at age 33 after controlling for childhood behaviour adjustment and BMI, and a number of demographic and epidemiological factors [16]. The current study explores the same data set which has recently had various important and neglected factors added such as personality traits and looks at BMI when cohort members were at 50 years.

 There is a literature on personality disorders and eating disorders/obesity which has implicated various disorders (i.e. OCPD) with specific eating disorders [17]. This study focused on normal personality traits and obesity. There has also been research meta-analysing 20 psychological studies on the associations between Conscientiousness and health [18]. Results showed that higher levels of Conscientiousness were significantly and positively linked to longevity. In recent years the association between personality traits, BMI, and obesity have been examined by two research teams [19,20]. One group examined a set of socioeconomic indices and personality traits in relation to adult obesity [19]. They found that, for both men and women, trait Conscientiousness was associated with adult obesity, and the effect of parental socioeconomic index was explained completely by adult socioeconomic index. Another group examined how personality traits were associated with adiposity and with fluctuations in BMI [20]. They found that participants higher on Neuroticism or Extraversion, or lower on Conscientiousness, had higher BMI, and high Neuroticism and low Conscientiousness were associated with weight fluctuations, measured as the variability in weight over time. Although a number of studies on obesity have used longitudinal birth cohort samples, no study has yet examined the associations between obesity and above mentioned socioeconomic, psychological, neurological, and behavioural factors *together as a whole*. Since many correlates of obesity are inter-correlated, it is important to ascertain the independent associations between these factors and obesity, so that the causes and correlations of obesity would be better understood, and initiatives and intervention programmes could be designed with more effective outcomes.

 There are a host of distal and proximal factors that influence BMI and obesity. Children’s parental social class influences their diet, education and behavioural adjustment. Further their birth weight and gestations are found to be associated with later development. Despite these predictive socioeconomic factors, behavioural and psychological factors like pathological symptoms were associated with recurrent binging and obesity in later years [4,17]. Thus, better educated, higher social class individuals, who are more conscientious, may be expected to lead a healthier life-style [18].

 The aims of the current study were threefold: First, to investigate whether childhood neurological function (poor physical control and coordination) would have a long lasting adverse effect (over 40 years) on adult obesity. This study extends the previous study [16] which provided evidence of the associations between childhood neurological function and adult obesity at age 33. We examined whether childhood neurological function would be significantly associated with adult obesity 17 years later at age 50, and whether this association would be significant and independent when a set of socioeconomic, psychological and behavioural factors were taken into account. The second aim of the study is to look at the association between physical exercise and adult obesity. Although in clinical setting the negative effect of physical exercise on obesity has well been documented, the association between physical exercise and obesity in community samples is less clear. None of the studies reviewed above included this factor, indeed a number of studies have suggested that physical exercise should be included in further investigations [15,16]. Third, in terms of the associations between personality factors and obesity, previous studies have not controlled childhood factors such as parental BMI and childhood BMI, childhood cognitive ability and behaviours, and factors in adulthood such as anxiety and depression, all of these were found to be associated with adult obesity [4,14-16]. It is not clear whether personality traits would be independently associated with obesity after taking into account the confounding factors. This study set out to examine this, using the longitudinal birth cohort data which have provided information on these factors.

It is hypothesised that 1) poor childhood neurological function would be significantly associated with adult obesity; 2) parental social class would significantly influence adult obesity; 3) education would be significantly associated with adult obesity; 4) personality trait Conscientiousness would be a significant predictor of adult obesity; 5) psychological distress would be significantly associated with adult obesity; 6) physical exercise would be significantly associated with adult obesity; and 7) all these six factors (neurological, social, psychological, and behavioural or physical) might be independently associated with adult obesity, after adjusting for birth weight and gestation, maternal and paternal BMI, childhood BMI, childhood cognitive ability and behavioural adjustment, and diet.

## Method

### Sample

The National Child Development Study 1958 (NCDS) is a large-scale multidisciplinary longitudinal study of the 17,415 individuals who were born in Great Britain in a week in March 1958 [21,22]. In the study participants were recruited as part of a perinatal mortality survey, and they have been followed up at 7, 11, 16, 23, 33, 42, 46, and 50 years. At age 50, 9,790 (response = 79.5%) participants from a target sample of 12,316 subjects provided information on their BMI. The analysis presented here is based on 5,921 participants with complete data on all the measures we were interested in: childhood neurological function (assessed at age 7), education (at age 33), occupational attainment, personality traits, psychological distress, and physical exercise (all measured at age 50). Attrition of the sample has resulted in a slight under-representation of those participants who are most disadvantaged, but the remaining sample is generally representative of the original sample, which is in itself representative of the general British population [23]. 

### Measures

1. Childhood measures: Parental social class at birth was measured by the Registrar General’s measure of social class (RGSC). RGSC is defined according to occupational status and the associated education, prestige or lifestyle [24], and is assessed by the current or last held job. Where the father was absent, the social class (RGSC) of the mother was used. RGSC was coded on a six-point scale (I= unskilled occupations to 6 = professional) [25]. Parental BMI was computed following the Metric Imperial BMI Formula kg/m^2^ (weight in kilograms/ height in meters² ).The neurological function variables (poor hand control, poor co-ordination, overall clumsiness) were reported by teachers when subjects were at age 7. Teachers were instructed to score 0=“Doesn’t apply” (if the description does not fit the child), 1=“Applies somewhat” (if it is a marginal case), 2=“Certainly applies” (if the child certainly fits the description). As the assessments of neurological function were based on teachers’ observation of pupils, the total score of the Bristol social adjustment guide with 150 items [26], was used as a control variable to reduce the possibility that deviant behaviour influenced the teachers’ perceptions of children. Other childhood control variables include the height and weight of the cohort members which were measured by trained medical staff when subjects were at age 7 (BMI was computed following the Metric Imperial BMI Formula kg/m^2^), and cognitive ability tests which were measured when subjects were at age 11 consisting of 40 verbal and 40 non-verbal items administered at school. This measure of childhood intelligence has been used in many explorations of this data bank though a more comprehensive measure is available [27].

2. Adulthood measures: BMI was computed following the Metric Imperial BMI Formula kg/m^2^. Obesity at 42 and 50 years defined as body mass index ≥ 30 according to World Health Organisation recommendation [28]. At age 33, participants were asked about their highest academic or vocational qualifications. Responses are coded to the six-point scale of National Vocational Qualifications levels (NVQ) which ranges from ‘none’ to ‘university degree’/equivalent NVQ 5 or 6. Cohort members also provided information at age 33 on their diet, reported consumption of chips, sweets or chocolates, fruits, and fresh salad. Data on current or last occupation held by cohort members at age 50 were coded according to the Registrar General’s Classification of Occupations (RGSC), described above, using a 6-point classification. Personality traits were assessed by the 50 questions from the International Personality Item Pool (IPIP) [29]. Responses (5-point, from “Strongly Agree” to “Strongly Disagree”) are summed to provide scores on the so-called ‘Big-Five’ personality traits: Extraversion, Emotionality/Neuroticism, Conscientiousness, Agreeableness, and Intellect/Openness. A preliminary analysis showed that only Extraversion and Conscientiousness had significant associations with obesity and were used in the study. Alpha was 0.73 for Extraversion and 0.77 for Conscientiousness in the study. The z scores were used for the study. Psychological distress was assessed at age 50 using Rutter Malaise Inventory [30]. It comprised of 9 items with Yes/No. The content includes major psychological disorders such as anxiety and depression and physical exhaustion. Examples of items are whether the participant “often feels miserable and depressed”, or “feels tired most of the time”. The Alpha was 0.79 in the study. The cut-off point was used for the analyses. At age 50 cohort members provided information on the frequency of their physical exercise. Responses were coded to the six-point scale (1= less often, 2= 2-3 times a month, 3= once a week, 4=2 or 3 days a week, 5=4 or 5 days a week, 6=every day). 

## Results

### Descriptive Analysis

Table 1 shows the characteristics of the study population according to prevalence of obesity at 42 and 50 years. The prevalence of obesity was greater at the age of 50 than at the age of 42, from 14.2% to 23.6%, and gender differences of this change were shown in Table 1.

**Table 1:** Characteristics of the study population according to prevalence of obesity at 42 and 50 years.

			Obesity at age 42	Obesity at age 50
	n	%	%	%
*Gender*				
Male	2994	50.6	14.9	24.6
Female	2927	49.4	13.5	22.6
*Parental social class at birth*				
Unskilled (V)	439	7.9	16.6	26.0
Partly skilled (IV)	688	11.7	16.7	31.7
Skilled manual (III)	2915	49.6	16.0	25.2
Skilled non-manual (III)	661	10.9	11.0	20.0
Managerial\tech (II)	912	15.0	9.6	16.4
Professional (I)	306	5.0	7.8	15.4
*Educational qualifications at age 33*				
No qualifications	429	6.7	21.7	36.1
CSE 2-5/equivalent NVQ1	673	10.5	19.0	26.4
O Level/equivalent NVQ2	2077	26.3	14.6	25.8
A level/equivalent NVQ 3	921	18.8	11.5	21.0
Higher qualification/equivalent NVQ4	970	33.1	14.8	23.3
University degree/equivalent NVQ 5, 6	851	4.7	7.8	12.8
*Own current social class*				
Unskilled (V)	122	2.3	21.3	28.7
Partly skilled (IV)	639	10.9	16.3	25.7
Skilled manual (III)	1057	18.8	16.7	27.1
Skilled non-manual (III)	1223	20.2	11.9	22.8
Managerial\tech (II)	2514	41.7	14.1	22.3
Professional (I)	366	6.0	9.3	19.7

### Correlational analysis

Pearson product-moment correlation analysis was conducted among the variables used in the study. Table 2 shows the results.

**Table 2:** Pearson product-moment correlations of gender, adult obesity, parental social class, childhood neurological function, educational qualifications, occupational levels, personality traits, psychological distress, and physical exercise.

	*Variables*	Mean (SD)	1	2	3	4	5	6	7	8	9	10	11	12
1.	Gender	.49 (.50)	—											
2.	Obesity age 50	.24 (.42)	-.024	—										
3.	Parental social class	3.31 (1.23)	-.024	**-.099**	—									
4.	Poor hand control	.20 (.47)	-.147	**.046**	-.064	—								
5.	Poor co-ordination	.12 ( .38)	-.084	**.030**	-.009	.389	—							
6.	Overall clumsiness	.12 ( .37)	-.129	**.098**	-.041	.372	.484	—						
7.	Educational qualifications	2.66 (1.44)	-.096	**-.119**	.320	-.144	-.070	-.093	—					
8.	Occupational levels	4.09 (1.21)	-.014	**-.047**	.211	-.111	-.079	-.097	.461	—				
9.	Extraversion	29.47 (6.58)	.078	**.022**	.028	-.040	-.056	-.030	.069	.120	—			
10.	Conscientiousness	34.01 (5.26)	.103	**-.079**	.021	-.098	-.082	-.078	.064	.088	.146	—		
11.	Psychological distress	1.26 (1.73)	.162	**.046**	-.031	.030	.038	.041	-.085	-.078	-.141	-.108	—	
12.	Physical exercise	4.25 (1.35)	.067	**-.073**	-.020	.008	.001	.005	-.074	-.082	.019	.034	.005	—

Note: Variables were scored such that a higher score indicated being female, a higher score on obesity, a more professional occupation for parents, highest educational qualification, more professional occupation, a higher score on Extraversion and Conscientiousness, a higher score on psychological distress, and a higher score on frequency of physical exercise.

Table 2 shows that parental social class, education, trait Conscientiousness, psychological disorder, and physical exercise were all significantly and negatively associated with adult obesity (r = -.05 to -.12, *p*<.001); whereas childhood neurological function variables were significantly and positively associated with adult obesity (r=.03, *p*<.05 to r=.10, *p*<.001). There were significant inter-correlations among these variables. Apart from the well known associations between parental socioeconomic status and adult educational and occupational attainment (r = .21 to r =.46, *p*<.001), the association between parental social class and poor hand control was r = -.06, *p*<.001, between parental social class and overall clumsiness was -.04, *p*<.01. The associations between childhood poor neurological function and educational qualifications were r = -.07 to r = -.14, *p*<.001; and the associations between childhood poor neurological function and adult occupational prestige were r = -.08 to r = -.11, *p*<.001. Thus hypotheses 1) to 6) were all confirmed.

### Regression analysis

To investigate whether parental social class, childhood neurological function, social, psychological, and behavioural factors were independently associated with adult obesity, a series of logistic regression analyses were conducted. Table 3 shows the results of the odds ratios (95% CI).

**Table 3:** Odds ratios (95% CI) for obesity at age 50, according to parental social class, childhood neurological function, educational qualifications, occupational levels, traits extraversion and conscientiousness, psychological distress, and physical exercise.

	Odds ratio (95% CI)	Odds ratio (95% CI)	Odds ratio (95% CI)	Odds ratio (95% CI)	*p*-value†
*Measures*	Model 1	^b^ Model 2	^c^ Model 3	^d^ Model 4	
Gender	0.80 (0.68, 0.95)**	0.83 (0.68, 1.01)	0.88 (0.72, 1.07)	0.83 (0.67, 1.03)	0.074
Parental social class	0.91 (0.85, 0.98)**	0.95 (0.88, 1.03)	0.96 (0.88, 1.04)	0.96 (0.88, 1.04)	0.306
Poor hand control	0.93 (0.77, 1.13)	0.94 (0.75, 1.19)	0.96 (0.76, 1.22)	0.97 (0.76, 1.23)	0.742
Poor co-ordination	0.80 (0.62, 1.04)	0.74 (0.54, .1.02)	0.77 (0.56, 1.07)	0.77 (0.55, 1.07)	0.116
Overall clumsiness	1.88 (1.49, 2.38)***	1.61 (1.21, 2.15)***	1.58 (1.17, 2.15)**	1.60 (1.18, 2.18)**	0.002
Educational qualifications	0.84 (0.79, 0.90)***	0.86 (0.79, 0.93)***	0.88 (0.81, 0.96)**	0.87 (0.80, 0.95)**	0.002
Own social class	1.00 (0.93, 1.08)	1.02 (0.93, 1.12)	1.02 (0.93, 1.12)	1.02 (0.93, 1.12)	0.699
Extraversion	1.16 (1.06, 1.26)***	1.14 (1.04, 1.26)**	1.15 (1.04, 1.27)**	1.15 (1.04, 1.27)**	0.006
Conscientiousness	0.87 (0.80, 0.95)***	0.84 (0.76, 0.93)***	0.83 (0.75, 0.92)***	0.83 (0.75, 0.91)***	0.000
Psychological distress	1.45 (1.13, 1.86)**	1.33 (0.99, 1.80)*	1.33 (0.97, 1.81)*	1.35 (0.99, 1.84)*	0.043
Physical exercise	0.86 (0.81, 0.91)***	0.85 (0.79, 0.91)***	0.84 (0.78, 0.90)***	0.83 (0.77, 0.89)***	0.000

Note: *p<.05; **p<.01;***p<.001. ^b^ Adjusted for birth weight and gestation, childhood BMI, maternal and paternal BMI, and teachers’ assessment of children’s behavioural adjustment; ^c^ Adjusted for all factors in model 2 and childhood intelligence; ^d^ Adjusted for all factors in model 3 and diet. † *P*-values of model 4 after adjusting for birth weight and gestation, childhood BMI, behavioural adjustment, and intelligence, maternal and paternal BMI, and diet.

Model 1 shows the results of the net associations between neurological, social, psychological, and behavioural factors and obesity at the age of 50 years; Model 2 shows the results of the above associations adjusted for birth weight and gestation, childhood BMI, maternal and paternal BMI, and teachers’ assessment of children’s behavioural adjustment; Model 3 shows the results of the above associations with controlling factors in model 2 and childhood intelligence; and Model 4 shows the results of the above associations with controlling factors in model 3 and diet. 

Parental social class, childhood poor neurological function, educational qualifications, traits Extraversion and Conscientiousness, psychological distress, and physical exercise as well as gender were all significantly and independently associated with adult obesity in Model 1 (*p*<.01 to *p*<.001). However the association between parental social class measured at birth and adult obesity was no longer significant once the controlling variables were entered into the equation. Gender was not significantly associated with adult obesity at the significance level p<.05. 

The final model shows that poor neurological function in childhood, educational qualifications, traits Consciousness and Extraversion, psychological distress, and physical exercise were all significantly and independently associated with adult obesity. 

## Discussion

This study investigated two differential *psychological* factors (namely personality and mental health), a set of *neurological* measures, a *behavioural* measure (namely physical exercise), as well as *socioeconomic* factors such as parental social class, educational level achieved and occupational attainment *together* as determinants of adult obesity. We controlled for parental BMI, childhood BMI, childhood intelligence and behavioural adjustment, and diet. It contributes to the research on obesity in demonstrating that poor childhood neurological function, education, traits Conscientiousness and Extraversion, psychological distress, and physical exercise are all significant and independent predictors of adult obesity; each has explained unique individual variability. It shows that poorer childhood neurological function has an adverse effect on adult obesity 43 years later, whereas higher educational qualifications, high Conscientiousness and low Extraversion, and higher frequency of physical exercise have the protective effects on adult obesity, taking into account a set of confounding factors. 

Parental social class was significantly and independently associated with adult obesity when all the other five social, psychological, and behavioural factors were taken into account. It seems that the effect of this factor on adult obesity, at this point, was not completely explained by socioeconomic factors such as parental educational qualifications and occupational attainment as found in the previous study [19,31]. The association between parental social class and adult obesity ceased to be significant once the controlling variables were entered into the equation. 

Although some childhood factors can influence adult obesity (poor childhood neurological function, parental BMI, lower parental socioeconomic conditions), the effect of childhood socioeconomic conditions measured by parental social class is almost entirely explained by childhood behavioural adjustment and intelligence, and adult behavioural factors, such as diet. Thus, adult obesity can only be explained in part by childhood conditions, and largely is associated with social, psychological, and behavioural factors in adulthood, which are not totally beyond one’s own control. 

 This study shows that poor neurological function in childhood has an adverse effect on educational achievement, which in turn, affects weight control in adulthood. It also shows that poor childhood neurological function is associated with parental socioeconomic conditions [16]. Interventions may take this into account, for example, by providing physiotherapy service to those children who have poor neurological function to reduce the negative impact of this condition in their adulthood.

 This study highlights the role of two personality factors in adult obesity. It looks at the effects of personality traits on adult obesity in addition to the effects of childhood neurological function, education, mental health, and physical exercise on adult obesity after controlling for a set of related factors (see Table 2). The results of the study show clearly the protective nature of trait Conscientiousness. Many studies have demonstrated relatively strong associations between trait Conscientiousness and a wide range of outcomes such as health, income, and relationship stability [18,32]. Conscientiousness is associated with dependability and reliability, responsibility and efficiency, but perhaps most relevantly, with self-discipline and ability to postpone gratification. Conscientious people tend to have higher aspirational levels as well as an ability to resist self-indulgence [33]. It seems clear why this easy to measure personality trait is related to weight control as well as a range of other health outcomes in adults.

Trait Extraversion was also related to obesity. Although extraverts are often more active than introverts they are clearly more sociable, gregarious, and outgoing. They also tend to be more impulsive and sensation seeking. They may therefore expose themselves to many more food temptations at social gatherings and feel less able to resist temptations. Whilst extraverts may have the advantage of social support, which has been shown to predict weight control, it also seems their spontaneity and lack of reflection would affect their attempts at moderating food intake. There are numerous papers in the therapy and training area which suggest that intervention is more effective if adapted to the personality and preferences of the clients [34-37]. 

 This study also shows the important role of physical exercise in weight control in adulthood. The results of this study show that among the variables in the study, physical exercise is associated with personality trait; participants who scored higher on Conscientiousness tended to have more physical exercise. Physical exercise is also associated with education and occupation; those who had higher social status tend to have less physical exercise than those who were at the lower social status levels. However, it is not clear what influences the frequency of physical exercise in adulthood. Future research is required to understand the role of exercise in adult obesity together with other factors.

### Limitation and suggestions for future research

Although our study is based on a birth cohort with representative sample, the attrition of respondents over time was greater among the socioeconomically disadvantaged groups. Our results may thus be a conservative estimate of the long term influence of social inequalities experienced during childhood. This study is based on a British cohort, and may not be representative internationally. Inevitably some on the measures of the variables we were interested in were of necessity brief and may therefore have reduced reliability. Furthermore, it would be very desirable to have had personality traits measured earlier as well as later, so that the stability and change of these traits in relation to the outcome variable could be investigated, though there is considerable evidence of the stability of personality over time.
